# Descriptive Epidemiology of Hemophilia and Other Coagulation Disorders in Mansoura, Egypt: Retrospective Analysis.

**DOI:** 10.4084/MJHID.2010.025

**Published:** 2010-08-13

**Authors:** Youssef Al Tonbary, Rasha ElAshry, Maysaa El Sayed Zaki

**Affiliations:** 1 Pediatric Medicine; 2 Clinical pathology, faculty of medicine, Mansoura University, Egypt

## Abstract

Hemophilia represent the most severe inherited bleeding disorder (INB), it’s thought to affect inviduals from all geographical areas in equal frequency. In Egypt which has a population of approximately (80million) consanguineous marriage are frequent, therefore autosomal recessive coagulation disorders reach a higher prevalence than in many other countries.

The primary aim of this study was to describe the epidemiological situation of hemophilia in Mansoura, Egypt, as based on retrospective analysis of clinical records Mansoura University Children Hospital between years 2000 and 2008. The second aim was to assess the orthopedic complications and occurrence of hepatitis C in those patients and relate this status to the type of replacement therapy received prior to the study.

The study included 72 children with hematological disorders registered from 2000 to 2008 in MUCH. The hemophilic patient was defined as a person with physician-diagnosed hemophilia A or B and a measured factor VIII or IX activity level of 30% or less. Persons with acquired inhibitors of FVIII or FIX excluded. Severity level was categorized as mild if the factor activity level was 6–30%, moderate if 1–5% and severe if <1% of normal.

The severe presentation represents the majority in 76.7% followed by moderate severity in 17.2%.The commonest IBDs was hemophilia A affecting 44 patients, followed by Hemophilia B affecting 15 patients. The rare types were Factor XI deficiency, Factor V deficiency, Factor VII deficiency and combined FVIII, FIX and FX deficiency. The commonest orthopedic manifestation needing therapy was found among hemophilia A representing 8.3%. Hepatitis C viremia detected by PCR was found in 11.1% of patients. The bleeding complications as hematoma or hemarthrosis were the common complications. Nevertheless, 44.4% of patients had no complications, From this study we can conclude that the most common IBDs in our locality is hemophilia A followed by hemophilia B. The common presenting symptom was bleeding following male circumcision. Hepatitis C infection and arthropathy represented the main complications. The discovery of IBDs in young age children with proper supportive therapy could prevent arthropathy. Proper screening of blood and blood products reduce the risk of viral hepatitis and HIV acquisition.

## Introduction:

Inherited bleeding disorders (IBDs) are caused by quantitative and qualitative alterations of either platelets or plasma proteins involved in coagulation and fibrinolysis.

Hemophilias are the most frequent IBD. The congenital bleeding disorders haemophilia A and B are estimated to affect between one in 10 000 and one in 50000 males.^1^

Studies of these diseases revealed that they result in varying degrees of bleeding diathesis. This deserves attention, not only to quantitative abnormalities but also to some IBDs, which reflect the synthesis of dysfunctional coagulation proteins or production of abnormal platelets.^2^

Hemophilias are the most frequent IBDs. However, von Willebrand disease (VWD) and platelet function defects (PFDs) are less common causes of bleeding. Various studies have reported that VWD is the most common congenital bleeding disorder in the population.^1^,^3^,^4^

In Egypt which has a population of approximately (80 million) consanguineous marriage are frequent, there fore autosomal recessive coagulation disorders reach a higher prevalence than in many other countries.

According to survey from the world federation of hemophilia (WFN) 80% of persons with hemophilia in the world are receiving minimal or no treatment at all and often do not survive to adulthood, recently mortality among people with hemophilia declined substantially, this decline is owed to increased availability of clotting factors concentrates for the treatment of life threatening bleeding episodes and the improved management provided by specialised hemophilia treatment centers.

The primary aim of this study was to describe the epidemiological situation of hemophilia in Mansoura, Egypt as based on retrospective analysis of clinical records at Mansoura University children hospital between 2000 and 2008. The hospital serve all east Delta region including(Demiatta, sharkia, Dakahlia governorates with approximatly 20000 children visit the hospital yearly complaninig of various general diseases. The second aim was to assess the orthopedic complications and occurrence of hepatitis C in those patients and relate this status to the type of replacement therapy received prior to the study.

## Patients and Method:

Pediatric patients complaining of hemophilia were recruited from hematology unit at Mansoura University children hospital (MUCH) from 2000 to 2008. Hematologists collected demographic characteristics, clinical history, and laboratory and treatment data together with long term complications. MUCH provides medical care to patients with hemophilia according to published guidelines.

The haemophilic patient was defined as a person with physician-diagnosed haemophilia A or B and a measured factor VIII or IX activity level of 30% or less. Persons with acquired inhibitors of FVIII or FIX excluded. Severity level was categorized as mild if the factor activity level was 6–30%, moderate if 1–5% and severe if <1% of normal. Data collected included place of residence, date of registration at hemophilia clinic, age at diagnosis and registration, type and severity of hemophilia and family history. Pedigree data were used to determine the number of affected relatives in the family. The patients were subjected to the following:

1. Thorough information of the history was taken including detailed questionnaire regarding mode of inheritance, the age of the onset of bleeding, duration of bleeding with its frequencies/year and how to stop (spontaneous, local or drug therapy), positive family history of similar bleeding condition, past history of blood transfusion and the nature of bleeding manifestation (bruising, purpura, echymosis, epistaxis, bleeding with dental procedures and bleeding per orifices) and history of drug intake or arthritis to exclude acquired disorders of bleeding. Careful clinical examination (chest, heart, abdomen, skin and musculoskeletal system), considering skin ecchymotic patches or bruising, and purpura.
2. Laboratory investigations and sample collection: laboratory investigations were undertaken in the clinical pathology laboratory of MUCH (Egypt). Venous blood samples from each patient were withdrawn after medical consent and divided into two tubes: first on EDTA for complete blood picture and blood grouping and second on Na citrate (32 g/l trisodium citrate, proportion of nine volumes of blood to one volume of citrate) to perform coagulation studies. Patients and controls samples were subjected to:
  - Complete blood picture (Cell Dyne 1600 Abbott; Dupont Co., Wilmington, Delaware, USA) and blood grouping with electron microscopic examination.
  - Coagulation studies that included:
    - INR
    - Partial thromboplastin time (PTT) (Behnk electronik coagulator 1. no.1921; Behnk Electronik, Norderstedt, Germany, PBS liquid plastin; Organics, Noisy le Grand, France);
    - Measurement of levels of factor VIII (FVIII:Ag) and factor IX Ag (F IX:Ag) to detect if it is found but not functioning by enzyme-linked immunosorbent assay (ELISA) (Asserochrome Diagnostics Stago, Gennevilliers, France);
    - Measurement of VWF Ag (VWF:Ag) level by Rocket immunoelectrophoresis technique (Helena, Beaumont, Texas, USA);
    - Measurement of factor VIII (FVIII:Ac) and factor IX activity (FIX:Ac) by one-stage assay method (STA-deficient VIII and IX); (iv) ristocetin cofactor (RICOF) as a test forVWF activity (VWF:Ac) using RICOF assay kit (Helena Biosciences, Sunderland, United Kingdom);
  - Complete virological profiles for each sample to detect hepatitis C specific IgG, Hepatitis B s antigen and anti core IgM and HIV specific immunoglobulin G. Positive cases for HCV IgG were subjected to nested polymerase chain reaction to determine active infection.

## Statistical analysis:

The statistical analysis of data done by using excel program and SPSS program (Statistical package of social science) version 10.

For qualitative data (frequency & proportion) chi – squire test was used.

For quantitative data (mean ±SD) t – test was used for comparison of two groups and one way anova was used for comparison of more than 2 groups.

N.B P is significant if ≤ 0.05 at confidence interval 95 %

## Results:

**Figure 1.**
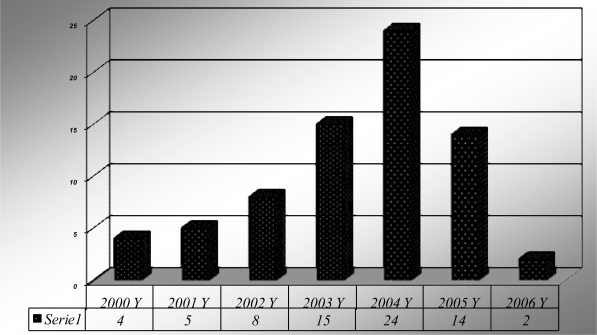
Distribution of cases during period from 2000 to 2008.

The study included 72 children with hematological disorders registered from 2000 to 2008 in MUCH. The registered number in 2004 was the larger one (24 patients) with rate decline after that, figure 1.

Table 1 summaries the severity of bleeding disorder among patients. The moderate presentation represents the majority in 17.2% followed by sever presentation in 4.7%, table 1.

The commonest IBDs was hemophilia A affecting 44 patients, followed by Hemophilia B affecting 15 patients. The rare types were the defect of Factor XI (hemophilia C), factor V deficiency, factor VII deficiency and combined factor VIII, IX and X deficiency (1 patient for each), table 2.

The mean± SD of duration hemophilia A was 2.7± 1.7 and for hemophilia B was 3.1± 1.4. Longer duration was noticed for other IBDs, table 3.

The commonest orthopedic manifestation needing therapy was found among hemophilia A representing 8.3%. In general this complications was uncommon among patients, table 4.

Hepatitis C viremia detected by PCR was found in 11.1% of patients, table 5.

The mean presenting symptoms was bleeding following male circumcision 51.4% followed by post traumatic bleeding in 36.1%. Accidental discovery was found to be common in female, table 6.

The bleeding complications as hematoma or hemarthrosis were the common complications. Nevertheless, 44.4% of patients had no complications, table 7.

## Discussion:

IBDs are caused by quantitative and qualitative alterations of either platelets or plasma proteins involved in coagulation and fibrinolysis. The severity is generally related to the degree of the underlying defect. Rapid and reliable identification of these diseases is important to allow the adoption of appropriate substitutive or supportive therapies. The study was formulated on the observation that in Egypt, genetic diseases do not receive any public health support because they are considered to be rare conditions with low prevalence. Furthermore, actual statistics on the demography of genetic diseases in the Egyptian population are largely unavailable. The purpose of this study was to generate base line epidemiological information on the status of hemophilia in MUCH. In the last 6 years increase in number of detecting of sever hemophilia in infancy indicating increased awareness among practitioners about hemophilia^5^

The present study reveals that the commonest IBDs was hemophilia A affecting 44 patients, followed by Hemophilia B affecting 15 patients.

For age presented at diagnosis, the older age was for VWD with range from 30 months up to 75 months, table 2.

The rare types were Factor XI, Factor V deficiency, Factor VII deficiency and combined F VIII, FIX and FX deficiency.

This result reflects that hemophilia is the most frequently encountered IBD and points to increased incidence of platelet dysfunction disorders. Few published studies in Saudi Arabia, Jordan and Egypt describe the distribution of IBD in the population.^6^,^7^,^8^ They obtained the distribution of IBD resembling what has already been established by western countries. They reported that VWD was the second most common cause of IBD with the exception of increased platelet disorders, mostly due to the increased rate of consanguinity in the community.

Also, there is mixing of population present in Mansoura city. More than seventy five per cent of the hemophilic patients had the severe type of the disease. This is somehow higher than results that obtained by other studies elsewhere.

Although our results may be due to some degree of under diagnosis of the less severe forms of hemophilia, but it may be related to different ethnic and racial groups too.

For age presented at diagnosis, the older age was for VWD disease with age range from 30 months up to 75 months. This could be attributed to milder course of the disorder with later presentation. While for hemophilia the majority were presented before 1 year old. The age at diagnosis was determined from the case papers. The data revealed that 21% (109/510) of severe hemophilia A patients had reported their first symptoms before they were 1 year old.^5^ The registered number in 2004 was the larger one (24 patients) followed by decline in the rate. So, analysis of number of new patients being registered in the state revealed that, although there was a cumulative increase in the number of registrations, the number of cases being registered annually showed a steady decline, indicating that recruitment of known hemophiliacs in the community was being achieved. Case referrals were primarily of severe hemophiliacs, indicating under diagnosis of moderate and mild cases.

The problems with management of hemophilia in developing countries and the priorities in establishing hemophilia services have been discussed.^9^,^10^ This study highlights that hemophilia services are needed during their infancy, and identifies some areas of intervention that would improve the treatment available to patients. The major area of intervention is the need to improve the diagnosis of hemophilia, because a large number of patients either lack diagnosis or are diagnosed after considerable delay. The need to extend hemophilia services as well as increase awareness not only among practitioners, but also among family members, becomes obvious. A second area of intervention, which is of minimum importance at the current time, is the need to collect genetic information from families. These data would form the basis for genetic counseling, a feature that currently has very low priority.

Hemophilic arthropathy is found to be uncommon in our patients. This may reflect that this complication is uncommon in young age of children. This may be due to high intensity of replacement therapy seems to account for satisfactory results of orthopedic status of severe hemophilic patients. This may also account for low rate of bleeding complications. however unavailability of lyophilized concentrates which can help in implement a program of prophylaxis in children with severe disease still the cause of increase the incidence of long term complication such as chronic synovitis or intracranial hemorrhage.

Before the introduction of substitution therapy the cause of deaths among hemophiliacs was the almost unevitable bleeding. The low number of deaths due to all types of bleeding, except cerebral hemorrhage, proves the improvement and the effectiveness of the provided care. Nowadays, hemophiliacs are growing older and survive to experience diseases of the elderly like cancer and ischemic heart disease.

However, these have not yet become the commonest causes of death because of the appearance of transfusion transmitted viral diseases which is a major co-morbid condition among patients with hemophilia who received non-virus-inactivated or insufficiently inactivated large-pool clotting factor concentrates or cryoprecipitate.^11^,^12^ No cases were detected having HIV+ test probably due to early exclusive use of domestic products and the overall scarcity of treatment.

In the present study, 11% of patients had hepatitis C and none of them had serological evidence of hepatitis B virus. Viral Hepatitis C infection was found in 11% of patients. Already from 1975, 77% of polytransfused Greek hemophiliacs were infected by the hepatitis B virus^13^ and 47% had abnormal liver function tests.^14^ In the present study 11% of patients had active viremia, giving cause to the fears expressed in previous studies.^15^,^16^ Additionally, since hemophiliacs infected by HIV, HBV and HCV are not uncommon,^17^ more deaths due to combined infections may be expected for the near future. The risk of hepatitis virus infection and for HIV was great if the non-inactivated factor-concentrates are used for patient treatment. In addition blood and blood products transfusion carry the same risk if the donor was in the open window phase i.e. before antibodies appearance. The safety of factor concentrate nowadays are being actively inactivated for hepatitis viruses and for HIV. For blood products it may be safe to encourage blood bank to implant molecular techniques as nuclear amplifications technique for screening of mini pool of blood and blood products for safety of blood transfusion.

None of our patients had evidence of hepatitis B infection. This is may be due to good vaccination program for children for hepatitis B virus adopted by Egyptian national service. A more strict policy for blood product usage, universal HCV screening and HBV vaccination is needed to abolish those disease in patients with hemophilia

## Conclusions:

From this study we can conclude that the most common IBDs in our locality is hemophilia A followed by hemophilia B. The common presenting symptom was bleeding following male circumcision. Hepatitis C infection and arthropathy represented the main complications. The discovery of IBDs in young age children with proper supportive therapy could prevent arthropathy. Proper screening of blood and blood products reduce the risk of viral hepatitis and HIV acquisition.

## Figures and Tables

**Table 1.** Severity of bleeding disorders among patients.

			**Sex**		**Total**
			male	female	
Severity	< 1%	count	4	1	5
		% of total	5.5%	1.3%	6 %

	1–5 %	count	14		14
		% of total	19.4%		19.4%

	6–30 %	count	51	1	53
		% of total	76.6%	1.3%	73%

Total		count	69	3	72
		% of total	95.8%	4.1%	100.0%

**Table 2.** Distribution of IBDs among patients.

	**N**	**Mean**	**Std. Deviation**	**minimum**	**maximum**

Hemophilia A	44	16.4284	14.2723	1.00	55.00
Hemophilia B	16	14.3750	11.4666	1.00	37.00
Hemophilia C VW factor	1	27.0000	-	27.00	27.00
	2				
Facor V	1	1.0000	-	1.00	1.00
Factor VII def	1	13.0000	-	13.00	13.00
Combined factor VIII IX. Xdef	1	52.0000	-	52.00	52.00
Total	72	16.3414	14.0436	1.00	55.00

**Table 3.** The duration (in months) of IBDS among patients.

	**N**	**Mean**	**Std. Deviation**	**minimum**	**maximum**

Hemophilia A	44	42.6023	45.8449	1.50	168.00
Hemophilia B	16	22.3513	44.7736	0.26	180.00
Hemophilia C	1	0.7000		0.70	0.70
VW factor	2	75.0000	63.6396	30.00	120.00
Facor V	1	42.0000		42.00	42.00
Factor V13def	4	42.0000	30.8707	2.00	72.00
Factor VII def	1	3.0000		3.00	3.00
Factor X def	2	91.5000	125.1579	3.00	180.00
Combined factor VIII IX. Xdef	1	60.0000		60.00	60.00
Total	72	39.4558	47.3786	.26	180.00

**Table 4.** Orthopedic manifestation among patients.

			Orthophtsio-Therapy		Total
			negative	positive	
Diagno2	Hemophilia A	count	38	6	44
		% of total	52.8%	8.3%	61.1%

	Hemophilia B	count	15	1	16
		% of total	20.8%	1.4%	22.2%

	Hemophilia C	count	1		1
		% of total	1.4%		1.4%

	VW factor	count	2		2
		% of total	2.8%		2.8%

	Facor V	count	1		1
		% of total	1.4%		1.4%

	Factor V13def	count	4		4
		% of total	5.6%		5.6%

	Factor VII def	count	1		1
		% of total	1.4%		1.4%

	Factor X def	count	2		2
		% of total	2.8%		2.8%

	Combined factor VIII IX. X def	count	1		1
		% of total	1.4%		1.4%

Total		count	65	7	72
		% of total	90.3%	9.7%	100.0%

**Table 5.** Viral Infections among IBDS.

			**Sex**		**Total**
			male	female	
Virolgy	Notdone	count	13		13
		% of total	18.1%		18.1%

	Hcv – Ve	count	47	4	51
		% of total	65.3%	5.6%	60.8%

	Hcv+Ve	count	7	1	8
		% of total	9.7%	1.4%	11.1%

Total		count	67	5	72
		% of total	93.1%	6.9%	100.0%

**Table 6.** Bleeding symptoms after diagnosis of IBDS.

			**Sex**		**Total**
			male	female	
Presentation Symptoms	Post-Circumcision	count	37		37
		% of total	51.4%		51.4%

	Orificialbleeding	count	4		4
		% of total	6.5%		5.6%

	Post Traumatic	count	26	5	31
		% of total	36.1%	6.9%	43.1%

Total		count	67	5	72
		% of total	93.1%	6.9%	100.0%

**Table 7.** Complications associated with IBDS.

			Sex				Total

			No Complication	Hemoarthrosis	Orifascialble eding	Others	
Diagno2	Hemophilia A	count	19	11	8	6	44
		% of total	25.4%	15.3%	11.1%	8.3%	61.1%

	Hemophilia B	count	7	5	2	2	16
		% of total	9.7%	6.9%	2.6%	2.6%	22.2%

	Hemophilia C	count				1	1
		% of total				1.4%	1.4%

	VW factor	count	1		1		2
		% of total	1.4%		1.4%		2.8%

	Facor V	count	1				1
		% of total	1.4%				1.4%

	Factor V13 def	count	3			1	4
		% of total	4.2%			1.4%	5.6%

	Factor VII def	count				1	1
		% of total				1.4%	1.4%

	Factor X def	count	1		1		2
		% of total	1.4%		1.4%		2.8%

	Combined factor VIII IX. Xdef	count			1		1
		% of total			1.4%		1.4%

Total	count		32	16	13	11	72
	% of total		44.4%	22.2%	18.1%	15.3%	100.0%
